# Genetic signature analysis of *Perkinsus marinus* in Mexico suggests possible translocation from the Atlantic Ocean to the Pacific coast of Mexico

**DOI:** 10.1186/s13071-017-2304-4

**Published:** 2017-08-02

**Authors:** Juan Pablo Ek-Huchim, Ma. Leopoldina Aguirre-Macedo, Monica Améndola-Pimenta, Victor Manuel Vidal-Martínez, Juan Antonio Pérez-Vega, Raúl Simá-Alvarez, Isabel Jiménez-García, Roberto Zamora-Bustillos, Rossanna Rodríguez-Canul

**Affiliations:** 1 Laboratorio de Inmunología y Biología Molecular, Centro de Investigación y de Estudios Avanzados del IPN (CINVESTAV-IPN) Unidad Mérida, Carretera Antigua a Progreso Km. 6, 97310 Mérida, Yucatán Mexico; 2 Laboratorio de Patología Acuática, Centro de Investigación y de Estudios Avanzados del IPN (CINVESTAV-IPN) Unidad Mérida, Carretera Antigua a Progreso Km. 6, 97310 Mérida, Yucatán Mexico; 3 Instituto Tecnológico de Boca del Rio, Carretera Veracruz-Córdoba Km. 12, 94290 Boca del Río, Veracruz Mexico; 4 Instituto Tecnológico de Conkal, Antigua Carretera Mérida-Motul Km. 16.3, 97345 Conkal, Yucatán Mexico

**Keywords:** *Perkinsus marinus*, *Crassostrea virginica*, Gulf of Mexico, Transfer, Mexican Pacific coast

## Abstract

**Background:**

The protozoan *Perkinsus marinus* (Mackin, Owen & Collier) Levine, 1978 causes perkinsosis in the American oyster *Crassostrea virginica* Gmelin, 1791. This pathogen is present in cultured *C. virginica* from the Gulf of Mexico and has been reported recently in *Saccostrea palmula* (Carpenter, 1857), *Crassostrea corteziensis* (Hertlein, 1951) and *Crassostrea gigas* (Thunberg, 1793) from the Mexican Pacific coast. Transportation of fresh oysters for human consumption and repopulation could be implicated in the transmission and dissemination of this parasite across the Mexican Pacific coast. The aim of this study was two-fold. First, we evaluated the *P. marinus* infection parameters by PCR and RFTM (Ray’s fluid thioglycollate medium) in *C. virginica* from four major lagoons (Términos Lagoon, Campeche; Carmen-Pajonal-Machona Lagoon complex, Tabasco; Mandinga Lagoon, Veracruz; and La Pesca Lagoon, Tamaulipas) from the Gulf of Mexico. Secondly, we used DNA sequence analyses of the ribosomal non-transcribed spacer (rNTS) region of *P. marinus* to determine the possible translocation of this species from the Gulf of Mexico to the Mexican Pacific coast.

**Results:**

*Perkinsus marinus* prevalence by PCR was 57.7% (338 out of 586 oysters) and 38.2% (224 out of 586 oysters) by RFTM. The highest prevalence was observed in the Carmen-Pajonal-Machona Lagoon complex in the state of Tabasco (73% by PCR and 58% by RFTM) and the estimated weighted prevalence (WP) was less than 1.0 in the four lagoons. Ten unique rDNA-NTS sequences of *P. marinus* [termed herein the “*P. marinus* (Pm) haplotype”] were identified in the Gulf of Mexico sample. They shared 96–100% similarity with 18 rDNA-NTS sequences from the GenBank database which were derived from 16 Mexican Pacific coast infections and two sequences from the USA. The phylogenetic tree and the haplotype network showed that the *P. marinus* rDNA-NTS sequences from Mexico were distant from the rDNA-NTS sequences of *P. marinus* reported from the USA. The ten rDNA-NTS sequences described herein were restricted to specific locations displaying different geographical connections within the Gulf of Mexico; the Carmen-Pajonal-Machona Pm1 haplotype from the state of Tabasco shared a cluster with *P. marinus* isolates reported from the Mexican Pacific coast.

**Conclusions:**

The rDNA-NTS sequences of *P. marinus* from the state of Tabasco shared high similarity with the reference rDNA-NTS sequences from the Mexican Pacific coast. The high similarity suggests a transfer of oysters infected with *P. marinus* from the Mexican part of the Gulf of Mexico into the Mexican Pacific coast.

## Background

The protozoan *Perkinsus marinus* (Mackin, Owen & Collier) Levine, 1978 (Phylum Perkinsozoa) is one of the main pathogens of the American oyster *Crassostrea virginica* Gmelin, 1971 [1], causing perkinsosis. This pathogen is listed as notifiable by the World Organization for Animal Health (OIE) (http://www.oie.int/en/animal-health-in-the-world/oie-listed-diseases-2016/). Perkinsosis is associated with high mortality of populations of *C. virginica* [1–5]. The distribution of *C. virginica* infected with *P. marinus* from the southeastern coast of the USA portion of the Gulf of Mexico to the central, and northern USA Atlantic seaboard is well documented. To date, *P. marinus* remains a threat to *C. virginica* populations in these regions [3, 6, 7]. This parasite also caused major economic losses after its accidental introduction into Pearl Harbor, Hawaii [8]. In Brazil, *P. marinus* has been found infecting the mangrove oysters *Crassostrea rhizophorae* (Guilding, 1828) [9–11] and *Crassostrea gasar* (Deshayes, 1830) [9]. *Perkinsus marinus* has also been detected in Panamanian waters infecting *C. virginica* and *C. rhizophorae* in the Caribbean canal and *C. columbiensis* (Hanley, 1846) from the Pacific coast [12]. In Mexico, *P. marinus* has been reported in *C. virginica* from the Mexican coast of the Gulf of Mexico [3, 13–16]. In 1992 *P. marinus* was alleged to cause mortality of *C. virginica* stocks from the state of Tabasco, but prevalence and intensity of infection in the Gulf of Mexico, vary with location and season whether assessed either by Ray’s Fluid Thioglycollate Medium (RFTM) assay or PCR [3, 13–15, 17, 18]. The RFTM assay is highly useful to determine infection level and is considered the gold standard technique by the OIE [19]. Additionally, the non-transcribed spacer (NTS) is used as an rDNA marker to identify and discriminate between *Perkinsus* species using PCR [20, 21]. The two tests can be used together to increase the chance of detecting perkinsosis.

*Perkinsus marinus* is prevalent in the Gulf of Mexico [3, 13–15], but there was no evidence for its presence in the Mexican Pacific coast until 2006 when *P. marinus* caused high mortality in *Crassostrea corteziensis* (Hertlein, 1951) in the state of Nayarit [21] and *Crassostrea gigas* (Thunberg, 1793) in the state of Sonora [22], from Mexico’s Pacific coast. These outbreaks showed that oyster species appear to vary in their susceptibility to *P. marinus*; its pathogenicity and virulence could be associated with DNA molecular variability. This hypothesis is supported by the finding that low and transient infections were found in *Saccostrea palmula* (Carpenter, 1857) in the same areas [7, 23–28].

The adverse ecological and financial effects of the introduction of a given disease into a new geographical area are always a cause for concern. Importantly, bivalves are both hosts and vectors of microparasites, including *P. marinus* [29]. The spread of perkinsosis with the transport of live oysters for repopulation, commercialization and aquaculture purposes has devastated native and cultured species causing severe epizootics [9].

The aims of this study were to evaluate the presence of DNA variants of *P. marinus* and to assess the infection parameters of *P. marinus* in four coastal lagoons in the Gulf of Mexico, where *C. virginica* commercialization is highly profitable. We also appraised the transfer connection between *P. marinus* isolates from the Gulf of Mexico and those reported in the Mexican Pacific by analysing DNA sequences from the NTS region.

## Results

### Infection parameters of *Perkinsus marinus* in lagoons from the Gulf of Mexico

Overall, the prevalence of *P. marinus* in the four lagoons was 57.7% (338 out of 586 oysters) using PCR and 38.4% (225 out of 586 oysters) using RFTM. PCR had a sensitivity of 93.8% and a specificity of 94.4%, while the RFTM showed 62.4% sensitivity and 64.8% specificity. Significant differences were observed by comparing both prevalences (Chi-square test, *χ* ^2^ = 194.98, *df* = 1, *P* < 0.0001). The highest prevalence was observed in the state of Tabasco (G-test, *G* = 20.04, *df* = 3, *P* = 0.00016 6). Infection intensities were characterized as light (1–10 hypnospores/field) to moderate infection (11–100 hypnospores/field). Weighted prevalence (WP) values less than 1.0 indicated mostly light infections (Table 1).

**Table 1:** Parameter values of *Perkinsus marinus* in *Crassostrea virginica*. Prevalence, sensitivity and specificity in each lagoon were assessed by Ray’s Fluid Thioglycollate Medium (RFTM) and Polymerase Chain Reaction (PCR). Weighted prevalence (WP) was addressed by RFTM

Lagoon	Oysters sampled	Prevalence (%)		Sensitivity (%)		Specificity (%)		WP	No. of DNA sequences	*P. marinus* haplotypes		Accession number
		RFTM	PCR	RFTM	PCR	RFTM	PCR			No. of haplotypes	Code (frequency)	
Términos	120	6.7	48.3	13.8	100.0	55.4	100.0	0.07	20	2	Pm18 (11)	KX581121
											Pm88 (9)	KX581120
Carmen-Pajonal-Machona	300	60.0	73.0	79.5	97.2	62.8	93.8	0.69	10	1	Pm1 (10)	KX581119
Mandinga	75	25.3	46.7	51.4	94.7	69.6	97.5	0.25	20	5	Pm1 (5)	KX581118
											Pm2 (5)	KX581117
											Pm3 (4)	KX581115
											Pm4 (3)	KX581116
											Pm10 (3)	KX581113
La Pesca	91	19.8	28.6	42.3	61.1	79.5	89.2	0.19	30	2	Pm27 (15)	KX581114
											Pm30 (15)	KX581112

### Términos Lagoon, Campeche

**Fig. 1:**
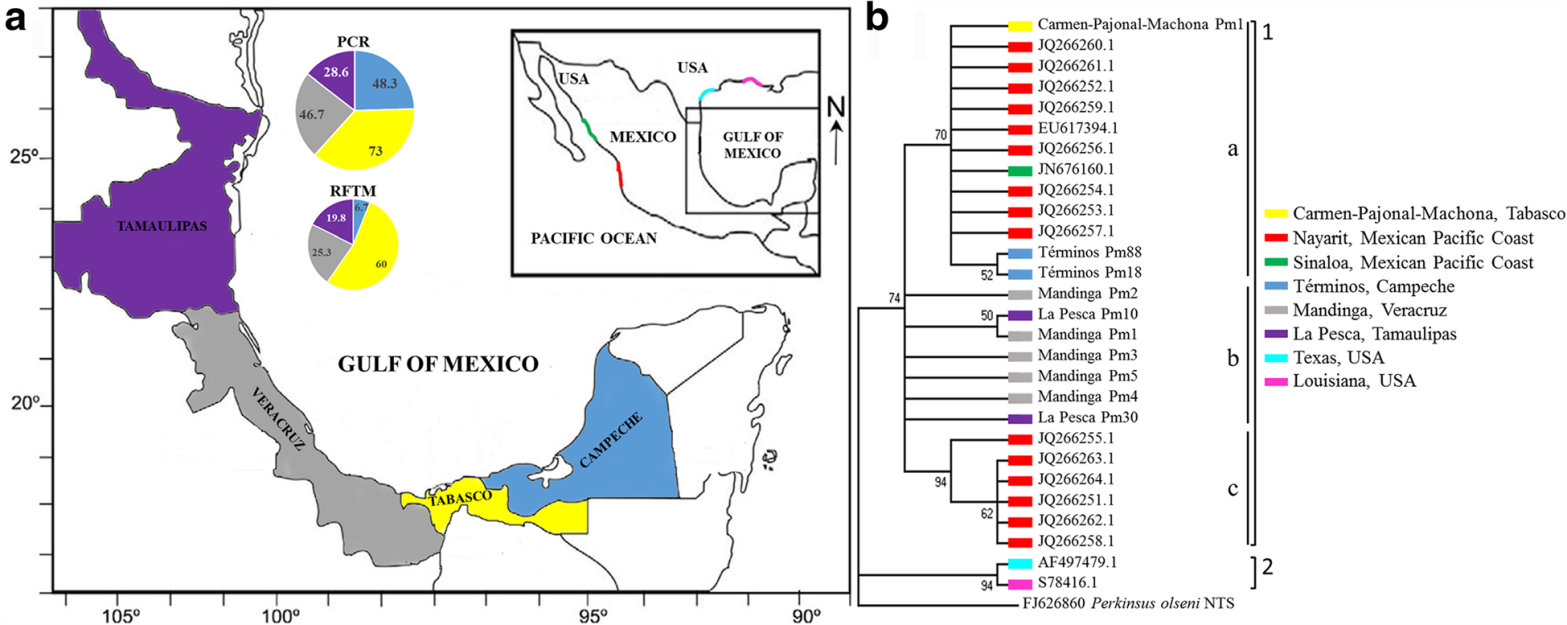
Study site locations from the Mexican Gulf of Mexico: **a** Términos Lagoon, Campeche; Carmen-Pajonal-Machona Lagoon Complex, Tabasco; Mandinga Lagoon, Veracruz; and La Pesca Lagoon, Tamaulipas. The prevalence by PCR and by RFTM is shown for each locality in the pie charts. **b** Phylogenetic tree inferred by the Maximum Likelihood method (3000 bootstrap replicates) using the 28 rDNA-NTS sequences of *Perkinsus marinus* in *C. virginica* from sampled lagoons of the Gulf of Mexico and the GenBank database; the phylogenetic tree with the highest log likelihood (-1922.1161) is shown. There was a total of 308 positions in the final dataset. A rDNA-NTS sequence of *P. olseni* was used as a root (FJ626860.1). Each colour corresponds to the geographical collection site as well as its haplotype. Red and green colours correspond to *P. marinus* isolates from the Pacific coast of Mexico. Light blue and pink colours correspond to *P. marinus* isolates from the USA

The prevalence was 48.3% (58 out of 120 oysters) using PCR and 6.7% (8 out of 120 oysters) using RFTM (Fig. 1). All organisms that were positive using the RFTM assay were positive by PCR, but 41.7% (50/120) of the oysters that were positive using PCR were negative by RFTM, and 41.7% (50/120) were negative for both tests (*χ* ^2^ = 9.16, *df* = 1, *P* = 0.0025). The sensitivity and specificity of PCR were both 100%, but using RFTM, they were only 13.8 and 55.4%, respectively. Infection intensity was light (1–10 hypnospores/field) in eight oysters, and the WP was 0.07 (Table 1).

### Carmen-Pajonal-Machona Lagoon Complex, Tabasco

The prevalence using PCR was 73% (219 out of 300 oysters), and it was 60% using RFTM (180 out of 300 oysters) (Fig. 1). In total, 58% (174/300) were positive by both tests, while 15% (45/300) were RFTM-negative but PCR-positive and 1.7% (5/300) were positive by RFTM but negative by PCR (*χ* ^2^ = 131.94, *df* = 1, *P* <0.0001). The sensitivity and specificity of the PCR method were 97.2% and 93.8%, respectively, and using RFTM; these values were 79.5 and 62.8%, respectively. Infection intensity was light (1–10 hypnospores/field) in 161 oysters, while in 4.3% (13/300), it was moderate (11–100 hypnospores/field), resulting in a WP of 0.69 for this lagoon (Table 1).

### Mandinga Lagoon, Veracruz

The prevalence using PCR was 46.7% (35 out of 75 oysters), and the prevalence was 25.3% (19 o ut of 75 oysters) using RFTM (Fig. 1). In total, 18 (24%) oysters tested positive by both tests. One oyster was positive by RFTM but negative by PCR, while the remaining 17 (22.7%) oysters were negative by RFTM but positive by PCR (*χ* ^2^ = 23.63, *df* = 1, *P* <0.0001). Sensitivity and specificity using PCR were 94.7% and 97.5% and using RFTM, these values were 51.4 and 69.6%, respectively. Infection intensity was light (1–10 hypnospores/field) in 19 oysters, and the WP for this lagoon was 0.25 (Table 1).

### La Pesca Lagoon, Tamaulipas

The prevalence according to PCR was 28.6% (26 out of 91 oysters), and according to RFTM, it was 19.8% (18 of 91 sampled) (Fig. 1). In all, 11 (12.08%) oysters were positive by both tests. Seven organisms were positive by RFTM but negative by PCR. The other 15 (16.5%) oysters were negative by RFTM but positive by PCR (*χ* ^2^ = 11.64, *df* = 1, *P* <0.0001). Sensitivity and specificity using PCR were 61.1% and 89.2%, respectively, and by RFTM, they were 42.3 and 79.5%, respectively. Infection intensity was light (1–10 hypnospores/field) in 18 oysters, with a WP of 0.19 (Table 1).

### Sequence data and phylogenetic analyses

The ten phylogenetic rDNA-NTS sequences of *P. marinus* [termed *P. marinus* (Pm) haplotype] found were submitted to GenBank. Two *P. marinus* rDNA-NTS sequence variants were from the state of Campeche (Términos Pm18 and Términos Pm88), one was from the state of Tabasco (Carmen-Pajonal-Machona Pm1), five were from the state of Veracruz (Mandinga Pm1, Mandinga Pm2, Mandinga Pm3, Mandinga Pm4 and Mandinga Pm5), and two were from the state of Tamaulipas (La Pesca Pm10 & La Pesca Pm30) (Fig. 1). Nucleotide variability, including deletions and insertions, was observed at 17 positions of the amplified 307 ± 1 bp fragment. Table 2 shows the nucleotide position in each rDNA-NTS sequence. These rDNA-NTS sequences had a maximum identity (96–100%) with 18 *P. marinus* rDNA-NTS sequences from GenBank. Thus, a total of 28 rDNA-NTS sequences were used for phylogenetic and haplotype network analyses.

**Table 2:** Nucleotide differences among ten rDNA-NTS sequences of *Perkinsus marinus* from the Mexican Gulf of Mexico: changes at 17 nucleotide positions are shown, including gaps, insertions, and substitutions

	Variation of nucleotide position																
Haplotypes	8	9	11	12	20	21	43	65	108	199	296	297	298	299	300	307	308
Carmen-Pajonal-Machona Pm1	A	T	–	G	C	A	C	G	T	A	G	A	G	A	T	A	A
Términos Pm18	A	A	T	T	C	A	C	G	T	A	G	A	A	T	T	A	A
Términos Pm88	A	G	T	–	C	A	T	G	T	A	G	A	A	T	T	A	A
Mandinga Pm1	A	T	–	G	C	T	T	A	T	A	G	A	G	A	T	A	A
Mandinga Pm2	T	T	T	G	C	A	T	G	T	A	C	A	G	A	T	A	A
Mandinga Pm3	A	T	–	G	C	T	T	A	T	A	G	A	G	A	T	A	G
Mandinga Pm4	A	C	–	G	C	T	T	A	T	A	G	A	G	A	A	A	A
Mandinga Pm5	T	C	–	T	C	T	T	A	T	A	C	G	A	T	T	T	A
La pesca Pm10	A	T	–	G	C	T	T	A	T	A	G	A	G	A	T	A	A
La pesca Pm30	A	T	–	G	G	T	T	A	G	C	G	A	G	A	T	A	A

Overall, the rDNA-NTS sequences of *P. marinus* from Mexico (sequences from the Mexican coasts of the Gulf of Mexico and sequences from the Mexican Pacific coast) (Clade 1) were observed in a tree branch that had a strong bootstrap support. Clade 1 included three groups (A, B, and C): group “A” included the rDNA-NTS sequences of Carmen-Pajonal-Machona Pm1, Términos Pm88, Términos Pm18 and ten rDNA-NTS sequences reported from the Mexican Pacific coasts (JQ266259.1–JQ266261.1, JQ266252.1–JQ266254.1, JQ266256.1, JQ266257.1 [30]; EU617394.1 [21], and JN676160.1 [31]). Group “B” included the rDNA-NTS sequences from the Gulf of Mexico; Mandinga Pm2, La Pesca Pm10, Mandinga Pm1, Mandinga Pm3, Mandinga Pm4, Mandinga Pm5 and La Pesca Pm30. Group “C” included six rDNA-NTS sequences reported from the Mexican Pacific coast: JQ266255.1, JQ266262.1–JQ266264.1, JQ266251.1, and JQ266258.1 [30] (Fig. 1). Clade 2 was formed by the rDNA-NTS sequences of *P. marinus* from the USA (AF497479.1 [20] and S78416.1) [17]).

The single-level AMOVA analysis for population genetic structuring revealed a highly significant and strong genetic structure among the sampled lagoons (F_ST_ = 0.45, *P* < 0.0001), with 45.17% of the total genetic variance explained by variation among populations and 54.83% by variation within populations. Regarding the pairwise F_ST_ differences among populations, values ranged from 0.241 to 0.673, and all comparisons were highly significant (*n* = 6 comparisons, *P* < 0.001) (Table 3).

**Table 3:** Pairwise estimates of F_ST_ among rDNA-NTS sequences of *Perkinsus marinus* from the Mexican Gulf of Mexico. F_ST_ estimates are shown below diagonals, and *P* values are shown above diagonals. Significant *P*-values are indicated by an asterisk

Haplotypes	La Pesca	Mandinga	Carmen-Pajonal-Machona	Términos
La Pesca	–	< 0.001*	< 0.001*	< 0.001*
Mandinga	0.241	–	< 0.001*	< 0.001*
Carmen-Pajonal-Machona	0.647	0.500	–	< 0.001*
Términos	0.481	0.324	0.673	–

### Haplotype network

**Fig. 2:**
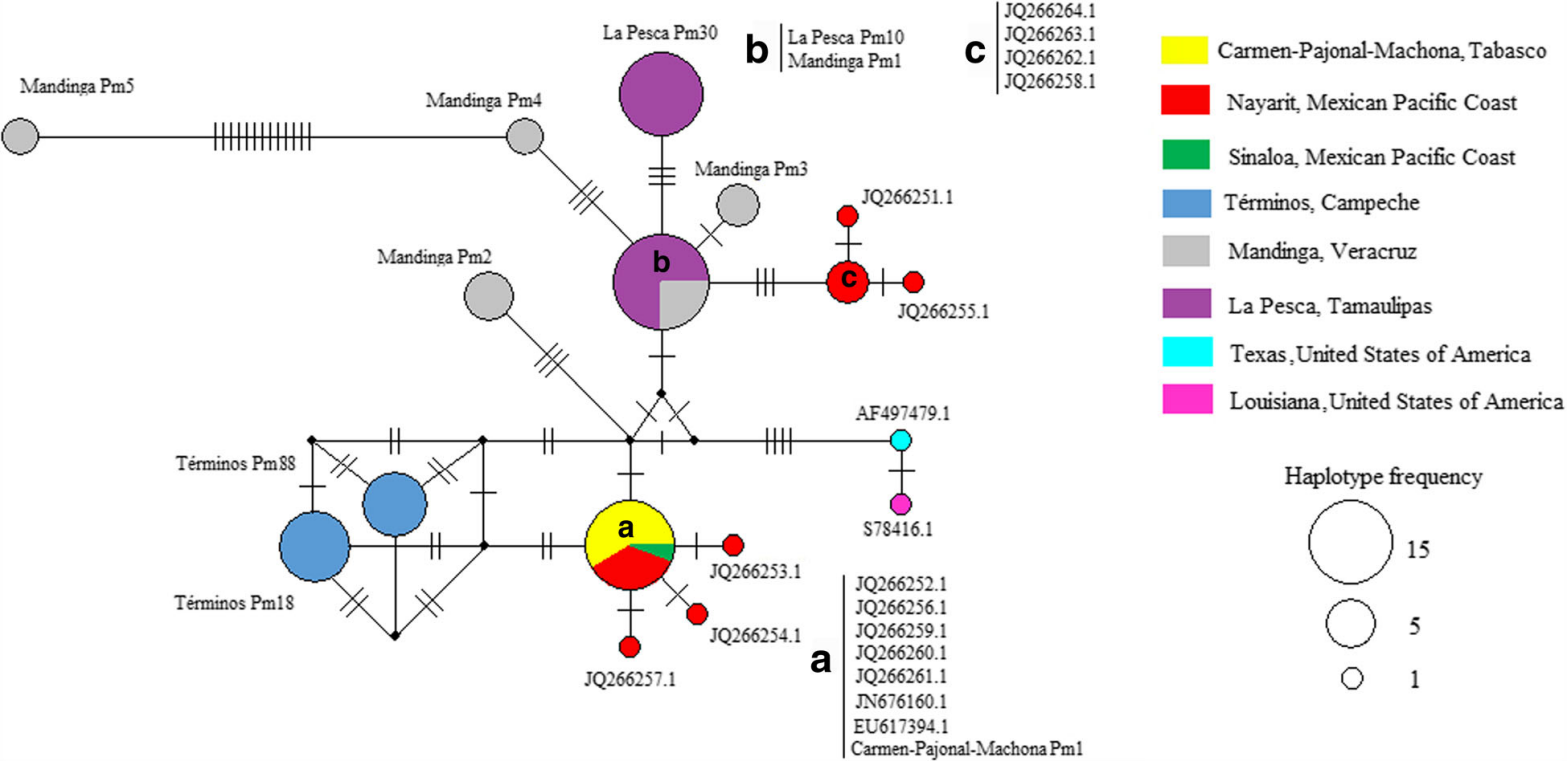
Haplotype network of 28 rDNA-NTS sequences of *P. marinus*. Circle areas are proportional to the number of observed rDNA-NTS sequences for each haplotype. Perpendicular lines represent the mutational steps differentiating haplotypes, and *small black circles* indicate the inferred median (unsampled haplotypes). Colours correspond to collection site geographical location (see Fig. 1): **a** *red* and *green haplotypes* correspond to the states of Nayarit and Sinaloa, respectively, from the Pacific coast of Mexico, the *yellow haplotype* is from the state of Tabasco in the Gulf of Mexico. **b** *grey* and *purple haplotypes* correspond to the states of Veracruz and Tamaulipas, respectively. **c** *red haplotypes* correspond to the state of Nayarit on the Pacific coast of Mexico. *Light blue* and *pink haplotypes* correspond to *P. marinus* isolates from the USA

In the haplotype network analysis (Fig. 2), sequences from the USA (AF497479.1 and S78416.1) were separated from the Mexican sequences. Four main haplogroups were observed: haplogroup A, formed by one haplotype from the state of Tabasco (Carmen-Pajonal-Manchona Pm1), included sequences from the Mexican Pacific coast (JQ266252.1, JQ266256.1, JQ266259.1–JQ266261.1 [30], EU617394.1 [21] and JN676160.1 [31]) and was closely related to the haplotypes from the Mexican Pacific coast (JQ266253.1, JQ266254.1 and JQ266257.1 [30]). Haplogroup B was formed from sequences from the state of Veracruz and Tamaulipas (Mandinga Pm1, La Pesca Pm10) and was closely connected with haplogroup C, which was formed exclusively from samples from the Mexican Pacific coast (JQ266262.1–JQ266264.1, and JQ266258.1) and related haplotypes JQ266251.1, JQ266255.1 [30]. The two haplotypes from the state of Campeche (Términos Lagoon) (Términos Pm18 and Términos Pm88) formed a separate haplogroup that was distanced from all other Mexican haplotypes. Mandinga Pm5 was the most differentiated haplotype; it was separated from the nearest haplotype (Mandinga Pm4) by thirteen mutational steps.

## Discussion

*Perkinsus marinus* impacts on the health and fitness of populations of the oyster *C. virginica* [1, 4, 29, 32, 33]. Consequently, it is crucial to determine the infection parameters (prevalence, abundance and infection intensity) of this parasite along its geographical distribution. In the lagoons of the Gulf of Mexico that were sampled in this study, the overall prevalence of *P. marinus* was 57.7% (338 out of 586 oysters) by PCR and 38.2% (224 out of 586 oysters) by RFTM. These differences in prevalence in lagoons from the Gulf of Mexico confirm previous studies [17, 34] that found that the PCR assay is more sensitive and specific than the RFTM assay to detect *P. marinus* in *C. virginica*. Robledo et al. [35] obtained similar results in the USA with the same set of primers. The RFTM assay has limitations in detecting low intensities of *P. marinus* infection, and diagnostic assessment is restricted only to trophozoites and hypnospores stages. In contrast, the PCR can detect low amounts of target DNA during all life stages of *P. marinus* [17, 34]. Both tests can be used to address a new infection in a given area [21], although the high sensitivity and specificity of the PCR test make it a valuable tool to address the prevalence of *P. marinus* in oyster production zones, especially for monitoring early infections [35]. Overall, light parasitic infection was detected in *C. virginica* stocks collected along the four lagoons from the Gulf of Mexico. However, in the Carmen-Pajonal-Machona Lagoon Complex in the state of Tabasco, low (1–10 hypnospores/40× field) to moderate infection intensities (11–100 hypnospores/40× field) with WP values less than one were observed. Scattered reports of mortality have been registered in this region [3], but in the present study, mortality was not quantified. Both tests were highly useful to address the infection. Histology was not used, but in previous research in the zone, this technique was a key factor to detect *P. marinus* infection [13, 14]. The OIE has stated that to declare a zone *Perkinsus*-free, it is necessary to use PCR, RFTM, and histology [36].

PCR was performed with specific primers designed from the non-transcribed spacer (NTS) region located between the 5S and 18S rRNA genes. This region is a highly variable domain, even between closely related species [20]. The ten unique rDNA-NTS sequences that composed the *P. marinus* (Pm) haplotype varied in 17 nucleotide positions and had a length of 307 ± 1 bp. They were similar to 18 rDNA-NTS sequences of *P. marinus* reported in GenBank. At least one DNA variant of *P. marinus* was detected in each lagoon. The major advantage of the PCR test used herein is that it can detect polymorphisms of *P. marinus* attributed to ploidy [37], increasing the possibility of detecting DNA variation due to the recombination of the NTS regions. *Perkinsus marinus* rDNA-NTS sequences analysed in this work strongly suggest restriction into specific locations, there for the population from each lagoon were genotypical diverse. Although F_ST_ values were significant to all lagoons, *P. marinus* from Tabasco state showed the highest differentiation (Table 3). The remaining rDNA-NTS variants occurred in different areas and exhibited different geographical connections (Fig. 2). The rDNA-NTS sequences from the USA (Clade 2) were on a different branch from all Mexican NTS sequences that had 94% bootstrap support (Clade 1). One rDNA-NTS sequence of *P. marinus* infecting *C. virginica* from the state of Tabasco exhibited 100% similarity with the rDNA-NTS sequences of *P. marinus* infecting native *C. corteziensis* from the state of Nayarit [21, 28, 30] and *S. palmula* from the state of Sinaloa, on the Pacific coast of Mexico [31]. The phylogenetic tree (Fig. 1) provides evidence of possible gene flow between the Gulf of Mexico and the Mexican Pacific coast. Also, haplotype network analysis revealed that one haplogroup formed entirely from rDNA-NTS sequences from the Mexican Pacific coast was closely related to haplotypes from the states of Veracruz and Tamaulipas, suggesting an alternative route of gene flow. Both phylogenetic tree (Fig. 1) and the haplotype network (Fig. 2) provide evidence of possible gene flow between the Gulf of Mexico and the Mexican Pacific coast.

Mexican oysters are primarily exported to the USA, and the states of Veracruz and Tabasco are the main producers [37]. Tabasco commercializes fresh oysters to other states in Mexico such as Puebla, Oaxaca, Chiapas and Veracruz [38]. There are no documented records of *C. virginica* introduction into the Pacific coast of Mexico from the Mexican Gulf of México, although Cáceres-Martínez et al. [21] recorded two routes of introduction of *C. virginica* to the northwest coast of Mexico. One route proceeds from the eastern coast of the USA via the state of Washington and another travel along the east coast of the USA. These authors suggested that *P. marinus* may have been introduced from these places to the Pacific coast. It has been well documented that transport of bivalves from one location to another for aquaculture purposes serves as a mechanical vector for parasitic transmission, contributing to pathogen distribution to regions with non-infected hosts [29, 39–43]. Transferring live oysters is a leading cause of disease outbreaks and epizootics [44].

Before 2006, there were no official reports of *P. marinus* in *Crassostrea* species or any other bivalve species along the Mexican Pacific coast. However, in July and August 2006, *P. marinus* caused massive mortality in farmed *C. gigas* in the Gulf of California (north-west Mexico) [22]. Moreover, between 2006 and 2014, the parasite was detected in cultured populations of native *C. corteziensis* oysters from the state of Nayarit [21, 28]. These outbreaks may have occurred due to lack of internal regulations that control the transport of aquatic organisms (i.e. *C. virginica*) from central Mexico [21, 45]. In subsequent years, *P. marinus* was detected in natural and cultured *S. palmula* populations in four coastal lagoons in the state of Sinaloa [31]. Despite the destructive effect of *P. marinus* in new environments and host species, no research has focused on the transfer of *P. marinus* from the Gulf of Mexico to the Pacific coast of Mexico.

The successful colonisation of a given parasite in its new environment varies with life-cycle and ability to transfer to local hosts, as well as with natural resistance and resilience in the new hosts and environment. Transport and cultivation of oysters have the potential to move organisms between sites [43]. Importantly, the range of parasitic expansion also depends on climate temperature and the genetic variability of the hosts, which regulates tolerance or resistance to the pathogen [4, 23]. The results from this study strongly suggest parasite transfer from the Gulf of Mexico to the Mexican Pacific coast, most likely via transportation of infected oysters from the state of Tabasco along the Gulf of Mexico.

In the Mexican Pacific coast, *P. marinus* caused high mortality in *C. gigas,* and high-intensity infections (~361 to 3,020,516 hypnospores g^-1^ tissue) were observed [22]. The detrimental effect of *P. marinus* in *C. gigas* could be associated with a DNA variant of the pathogen and the differential susceptibility of *C. gigas* [46–49]. In the north-eastern coast of the USA, there was evidence of a positive correlation between the increase in death of *C. virginica* stocks and a rise in the prevalence of *P. marinus* [39]. According to the results of the present study, the low to moderate infection intensities observed in *C. virginica* from the Gulf of Mexico could suggest that this parasite’s host pathogenicity may be declining in its natural host. However, when transferred to another environment, the pathogenicity increased, as indicated by the high parameters of infection and high mortality described in the Mexican Pacific coast [31, 50]. The *P. marinus* haplotype found in the state of Tabasco (Carmen-Pajonal-Machona Pm1) was identical to rDNA-NTS sequences in other host species from the Mexican Pacific coast. This result also supports the hypothesis that *P. marinus* infection is transient in the Gulf of Mexico (based on the low parasitic infections reported here). The same haplotype was detrimental in the Mexican Pacific coast, based on allegations of mortality and pathogenicity in their new hosts [28]. However, more work must be done to test these preliminary observations.

## Conclusions

Low to moderate *P. marinus* infection intensities were found in *C. virginica* from the four coastal lagoons along the western and southern coasts of the Gulf of Mexico. PCR was more efficient in detecting cases of *P. marinus* than the RFTM assay. Ten unique *P. marinus* rDNA-NTS sequences were detected restricted into each specific locations suggesting different populations within subregions (i.e. Tamaulipas and Veracruz), and the remaining rDNA-NTS variants that occurred at different places exhibited different geographical connections. Also, the sequence from the state of Tabasco (Carmen-Pajonal-Machona Pm1) had high similarity to rDNA-NTS sequences from the Mexican Pacific coast. Based on the *P. marinus* DNA molecular NTS variants, we provided information related to the transferal of *P. marinus* to new geographical areas (i.e. Mexico’s Pacific coast), in native (*C. corteziensis* and *S. palmula*) and introduced (*C. gigas*) oyster species from the Pacific coast of Mexico. This transfer was most likely anthropogenic. Thus, effective regulations are needed to prevent further introduction of notifiable diseases that could potentially expand and devastate non-infected areas and hosts of ecological and economic importance.

## Methods

### Sampling sites and oyster collection

A total of 586 *C. virginica* oysters were collected from four coastal lagoons along the western and southern coasts of the Gulf of Mexico, including the states of Campeche, Tabasco, Veracruz and Tamaulipas (Fig. 1). In March 2005, 120 oysters were collected from Términos Lagoon, Campeche and 300 from the Carmen-Pajonal-Machona Lagoon Complex, Tabasco. In May 2008, 75 oysters were gathered from Mandinga Lagoon, Veracruz and 91 oysters from La Pesca Lagoon, Tamaulipas.

The oysters were collected either by snorkelling or with the aid of racks. These organisms were transported fresh and alive to the Aquatic Pathology and Molecular Biology Laboratory, CINVESTAV-Mérida. Ten *P. marinus*-free oysters were obtained from the Hog Island Oyster Company (Marshall, California, USA) and used as negative controls; the negative samples were confirmed by PCR and RFTM of tissue.

After transport to the laboratory, each oyster was dissected with sterile forceps and scissors. Necropsy tools were rinsed and flame-sterilized using 96% ethanol between sample collections to prevent cross-contamination during sampling. A portion of the rectum, mantle, and gills of each organism was dissected and divided into two portions, each including fragments of these three organs. One portion was incubated in RFTM, and the other portion was preserved in 96% ethanol for DNA extraction. The infection intensity and weighted prevalence (WP) were determined by RFTM. Prevalence was reported as the percentage of positive organisms based on either RFTM or PCR (see below).

### Ray’s fluid thioglycollate medium (RFTM) assay

Tissues were incubated in RFTM for five days at room temperature in darkness, followed by staining with 0.5% Lugol’s iodine. Infection with *P. marinus* was indicated by blue-black pre-zoosporangia (hypnospores, usually 30–80 μm in diameter, observed at 40×) (see Ray [32]) for details of technique). The Office International de Épizooties (OIE) considers this procedure to be the gold standard for identification and surveillance of *Perkinsus* species [36, 51].

The infection intensity interpretation using the RFTM assay was performed according to Burreson et al. [1, 3]. Infections were ranked as negative (0 hypnospores/field), light (1–10 hypnospores/field), moderate (11–100 hypnospores/field) and heavy (> 100 hypnospores/field) [52] and assigned ratings of 0, 1, 3 and 5, respectively, for the calculation of weighted prevalence (WP) [53]. The prevalence determined by RFTM was used to calculate the WP, which combines prevalence and intensity into a single expression and is determined by adding the individual assigned values and dividing by the number of oysters sampled [54]. WP values less than 1.0 indicate mostly light infections, and values greater than 2.0 indicate high prevalence and severe infection (see Burreson et al. [3]; Aguirre-Macedo et al. [13]; Lassudrie et al. [55]).

### Polymerase chain reaction (PCR)-based assay

Genomic DNA was extracted from ~30 mg of the fixed material using a Wizard Genomic DNA Purification Kit (Promega, Madison, USA). The *P. marinus*-specific PCR was run using the primer set 300F (5′-CAC TTG TAT TGT GAA GCA CCC-3′), and 300R (5′-CAG TAA ACC TCT ACA GTG GTT-3′) [17], which were designed within the NTS region between the 5S and 18S rRNA genes. The PCR reactions were performed in a total volume of 25 μl containing 1 μl genomic DNA (20 ng), 0.2 mM dNTP mixture (Promega), 1× reaction buffer (50 mM KCl, 10 mM Tris-HCl [pH 9.0], 0.1% Triton X-100), each primer at 1 pM, 0.3 U *Taq* DNA polymerase (Promega) and 2.5 mM MgCl_2_.

Amplification conditions were initial denaturation at 91 °C for 3 min, 27 cycles with a denaturation step at 91 °C for 1 min, annealing at 58 °C for 1 min (increasing 1 s/cycle), and extension at 72 °C for 1 min (increasing 2 s/cycle); and a final extension cycle was 72 °C for 10 min. The PCR products were observed on 2% agarose gels stained with 2% ethidium bromide and using a 100-bp DNA ladder as a reference (Promega). A band at 307 bp was considered a positive result for *P. marinus* infection [35]. A negative control (DNA from *P. marinus*-free *C. virginica* tissues) was used in all assays. Cross-contamination was avoided by individually processing each sample under sterile conditions in a laminar flow cabinet. Prevalence was considered the percentage of oysters with a positive band at 307 bp (see Bush et al. [56].

### Statistical analysis

Chi-square (2 × 2) test with Yates’ correction and two-tailed Fisher’s exact tests using 95% confidence intervals (CI) were used to evaluate differences in the proportion of infection [57]. The significance of the differences in infection prevalence between lagoons was assessed using G-tests [58], with significance established at *P* = 0.05. Sensitivity and specificity were calculated in the same conventional chi-square test, considering as RFTM as a gold standard test. Sensitivity (also called the true positive rate). Sensitivity measures the proportion of positives that are correctly identified as such and were calculated using the formula: Sensitivity = TP/TP + FN. Specificity measures the proportion of negatives that are correctly identified as such and was calculated with the formula: Specificity = TN/TN + FP. True positives (TP) were those RFTM-, and PCR-diagnosed as positive. False positives (FP) were those RFTM diagnosed as negative but PCR as positive. False negatives (FN) were those RFTM diagnosed as positive, but PCR as negative. True negatives (TP) were those RFTM, and PCR diagnosed as negative [59, 60]. The analyses were performed using STATISTICA 8 software of Stat Soft, Inc. 1984–2007.

### Sequencing and phylogenetic analysis

DNA sequences were obtained using an automatic sequencer (Applied Biosystems, Mod. ABI 310) [61]. They were then analysed and edited with the Chromas Pro V.1.2 program (Technelysium Pty. Ltd., 2009) and aligned with Clustal X (2.0.12) software [62]. Unique *P. marinus* rDNA-NTS sequences [termed *P. marinus* (Pm) haplotype] were compared to DNA sequences from GenBank (https://www.ncbi.nlm.nih.gov/ncbisearch) to determine homology. Total rDNA-NTS sequences were used to construct the phylogenetic tree. The statistical model most suited to measuring divergence between rDNA-NTS *P. marinus* sequences obtained in this study and homologous rDNA-NTS sequences from the GenBank were assessed using Model Test 3.7 software [63]. Divergence was estimated using the Maximum Likelihood method under the General Time Reversible model with 3000 bootstrap randomizations [64]. A heuristic search tree was automatically generated using the Neighbour-Joining and BioNJ algorithms, which created a pairs distance matrix that was estimated using the Maximum Composite Likelihood (MCL) method. The topology, with the superior log likelihood value selected, was used to analyse all DNA sequences, including gaps and deletions. *Perkinsus olseni* was used as root (FJ626860.1). Statistical analyses (95% confidence intervals) used in tree construction were performed using MEGA 7 software [65]. Arlequin 3.5 software [66] was used to estimate population differentiation, calculating the pairwise F_ST_ values among sampled populations (coastal lagoons), and performing a hierarchical analysis of molecular variance (AMOVA) [67]. Significance values were calculated using 10,000 random permutations.

### Haplotype network

Relationships between *P. marinus* haplotypes and base pair changes were observed by constructing a haplotype network using the same rDNA-NTS sequences from *P. marinus* identified here and in the GenBank database. The haplotype network was calculated using the Median-Joining algorithm, applied with the NetWork v.4.6.1.2 software [68].

## Abbreviations

NTS: non-transcribed spacer
PCR: polymerase chain reaction
Pm: *Perkinsus marinus*
rDNA: ribosomal DNA
RFTM: Ray’s fluid thioglycollate medium
WP: weighted prevalence
